# The shaping of social perception by stimulus and knowledge cues to human animacy

**DOI:** 10.1098/rstb.2015.0075

**Published:** 2016-01-19

**Authors:** Emily S. Cross, Richard Ramsey, Roman Liepelt, Wolfgang Prinz, Antonia F. de C. Hamilton

**Affiliations:** 1 Wales Institute for Cognitive Neuroscience, School of Psychology, Bangor University, Wales, The Netherlands; 2 Department of Social and Cultural Psychology, Donders Institute for Brain, Cognition and Behaviour, Radboud University Nijmegen, The Netherlands; 3 Institute of Psychology, Westfällische Wilhelms-Universität, Münster, Germany; 4 Max Planck Institute for Human Cognitive and Brain Sciences, Leipzig, Germany; 5 Institute for Cognitive Neuroscience, University College London, London, UK

**Keywords:** social cognition, social robotics, animacy, fMRI, action observation network, mentalizing

## Abstract

Although robots are becoming an ever-growing presence in society, we do not hold the same expectations for robots as we do for humans, nor do we treat them the same. As such, the ability to recognize cues to human animacy is fundamental for guiding social interactions. We review literature that demonstrates cortical networks associated with person perception, action observation and mentalizing are sensitive to human animacy information. In addition, we show that most prior research has explored stimulus properties of artificial agents (humanness of appearance or motion), with less investigation into knowledge cues (whether an agent is believed to have human or artificial origins). Therefore, currently little is known about the relationship between stimulus and knowledge cues to human animacy in terms of cognitive and brain mechanisms. Using fMRI, an elaborate belief manipulation, and human and robot avatars, we found that knowledge cues to human animacy modulate engagement of person perception and mentalizing networks, while stimulus cues to human animacy had less impact on social brain networks. These findings demonstrate that self–other similarities are not only grounded in physical features but are also shaped by prior knowledge. More broadly, as artificial agents fulfil increasingly social roles, a challenge for roboticists will be to manage the impact of pre-conceived beliefs while optimizing human-like design.

## 1. Introduction

Detection and recognition of other agents is a necessary ability across species. It is an integral pre-requisite for social interaction: one must accurately identify others in order to appropriately interact with them. For instance, one would not expect a robot to offer the same opportunities for social interaction as a human. Considering the predicted rise of artificial agents in society performing tasks alongside humans in hospitals, care homes and schools [1], it will become increasingly important to distinguish between animate agents (e.g. humans) and inanimate agents (e.g. robots). Robots can act in the world by moving and achieving goals, but they are not sentient or intentional. Indeed, a key factor for classifying other agents is the perception of animacy—the presence of life in others. The distinct way that robots and humans look and move as well as what we know about their origins offer important cues to animacy [2]. As such, a key question for social cognition and social neuroscience research pertains to understanding the cognitive and neurobiological mechanisms that enable us to recognize animacy in other agents [3].

### (a) The neuroscience of social perception and cognition

**Figure 1.**
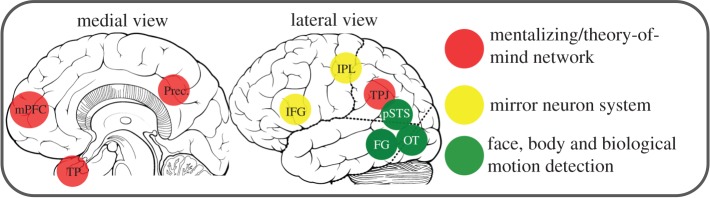
Social brain circuits. mPFC, medial prefrontal cortex; TP, temporal poles; Prec., precuneus; IFG, inferior frontal gyrus; IPL, inferior parietal lobule; TPJ, temporoparietal junction; pSTS, posterior superior temporal sulcus; FG, fusiform gyrus; OT, occipitotemporal cortex. The mirror neuron system and pSTS form the key nodes of the action observation network.

The neuroscience of social cognition is concerned with how the brain manages social interactions with others [4]. Several distinct brain circuits have been identified that process elements of our social worlds, three of which are of particular relevance to the current study (figure 1). Person perception research has shown how sensory systems are sensitive to the presence of conspecifics in the environment [5]. For instance, patches of cortex in the ventral visual stream including fusiform and occipitotemporal gyri respond preferentially to images of social stimuli (faces and bodies) compared to non-social stimuli (houses and cars) [6,7]. Accumulating evidence suggests the ventral visual stream contributes to understanding identity through processing physical appearance, such as facial features, body shape and posture [5,8].

Another form of social perception involves observing others moving through the environment and interacting with objects. Brain regions responding to the observation of others in action include posterior temporal gyri, inferior parietal lobule and inferior frontal gyrus [9–11]. The frontal and parietal responses are consistent with research into the mirror neuron system discovered in monkeys, which shows similar responses to performed and observed actions [12]. One dominant theory argues that this frontoparietal network enables action understanding through simulation by mapping observed actions onto the observer's own motor system [13].

Simply coding the physical characteristics of other agents and their movements would not, however, be sufficient to understand the meaning of their actions. It is also necessary to make inferences about information one cannot see, such as others' beliefs, desires, attitudes and traits [14]. A third strand of social cognition research—mentalizing—aims to delineate the cognitive and brain systems integral to representing such mental states of others [15]. Brain circuits spanning the medial prefrontal cortex (mPFC), temporoparietal junction (TPJ), temporal poles and precuneus are consistently engaged when inferring and evaluating mental states and are collectively known as the theory of mind network [4,15]. The ability to draw inferences about underlying intentions helps us to predict what another individual may do next and helps to regulate social interactions [3,16]. Together, the studies highlighted in this section have identified discrete brain circuits that subserve aspects of social perception and interaction. It is less clear, however, how social information is organized beyond a social–non-social distinction.

### (b) The ‘like-me’ hypothesis

One dominant model in social cognition states that understanding the similarity between self and other is a basic principle of social cognition and that humans have developed to seek out self–other equivalence [17,18]. This account, known as the ‘like-me’ hypothesis, further proposes that actions performed by oneself and another are represented in common cognitive codes [17]. At the core of the ‘like-me’ hypothesis is the proposal that cognitive and brain mechanisms have been shaped to show sensitivity to information that is physically or cognitively similar to one's own makeup. This view is consistent with the biological imperative to detect similar others as a foundation for successful navigation of the world [3].

One approach to test predictions that follow from the ‘like-me’ hypothesis has been to vary cues to human animacy. In such studies, the idea is that the more human-like an agent is perceived, in terms of physical appearance and intentionality, the more it is considered to be ‘like me’. These studies have fallen into two main camps based on the type of cues to human animacy under investigation. One camp has manipulated stimulus features, such as what an agent looks like or how it moves. The second camp has manipulated knowledge cues to animacy, such as whether an observer believes an agent to be human or not. Both cue types are of clear relevance to the study of social perception. Humans move in a particular way, for instance using a minimum jerk trajectory, and have a particular form (i.e. head above a torso with limbs). Such distinctive physical features can be diagnostic of a human presence. Likewise, knowledge cues also matter for interpreting human animacy. If you know the gorilla across the street is actually a man in a costume, your perception of the social environment would be markedly different from if you were not aware of this fact. In the following, we review behavioural and brain-imaging studies that have manipulated stimulus cues and knowledge cues to human animacy. Instead of an exhaustive review of all studies exploring animacy detection, our focus is on brain systems that index the distinction between human and non-human agents.

### (c) Stimulus cues to human animacy

The majority of research into cues influencing animacy perception has focused on stimulus cues to human animacy, such as what an agent looks like and how it moves. These can be considered ‘bottom-up’ cues that are determined by the visual appearance of the form and motion of an agent. Many studies have investigated responses along the ventral visual stream to depictions of human compared to non-human stimuli, such as other animals or inanimate objects [19,20]. Less research in the domain of person perception has varied cues to human animacy by comparing human to less human or robotic agents [21,22]. Gobbini *et al*. [21] showed similar engagement of core face perception areas—fusiform face area (FFA), occipital face area (OFA) and posterior superior temporal sulcus—when observing human and artificial, robotic faces. In addition, core face and body processing regions also respond to cartoon and schematic depictions of faces and bodies [6,22]. Thus, the ventral visual stream appears to be indifferent to animacy cues that are based on physical form and responds to real faces and bodies as well as face- and body-like forms.

In the domain of action perception, where agents are moving in the world and sometimes interacting with objects, results are mixed. The superior temporal sulcus has been shown to respond to biological motion, even in the absence of a clear human form [23,24]. Many studies have also compared the observation of actions performed by humans and robots. A common result is more engagement of sensorimotor brain regions collectively termed the action observation network (AON) and facilitated behavioural responses when the agent is more human than not [25,26]. For example, observing human form and motion increased motor priming in an imitation task [27,28]. In addition, right premotor cortex is engaged more during the observation of reaching actions performed by a human hand compared to a robotic claw [29]. These results are consistent with a self-similarity bias and more AON engagement when an observed agent is more human.

On further inspection of the action perception literature, however, several studies show indifference in the AON to degrees of stimulus-driven humanness or even a preference for non-human stimuli. For instance, Gazzola *et al*. [30] failed to find any difference in brain responses when participants viewed actions performed by a human or robotic hand. Likewise, Ramsey & Hamilton [31] found that the left anterior intraparietal sulcus, a core AON node, responded in a similar manner when participants observed a geometric shape or a human hand perform goal-directed actions. Moreover, some studies show an even greater response in the AON when perceiving non-human compared to human visual cues [32,33]. In two experiments, Cross and co-workers show greater engagement when watching rigid robotic movement compared to natural free-flowing dance moves that are more consistent with a human's motor repertoire [32]. This robust AON engagement was seen when participants observed a human actor dancing and when observing a robot toy animated to move in a similar manner. Therefore, the AON was shown to be more sensitive to rigid, non-human-like movement irrespective of animacy cues based on physical form. Finally, Saygin & Stadler [33] found that middle temporal gyrus and intraparietal sulcus are more sensitive to an android (a robot dressed as a human) than a clearly presented human or robot actor. Thus, the role of the AON in response to varying stimulus cues to human animacy remains somewhat unclear.

Stimulus cues can also drive mental state reasoning and engagement of the person knowledge or theory of mind network. Heider & Simmel [34] showed that when people observe simple shapes moving around as if they are interacting, they ascribe human-like mental states to these shapes. Using the same stimuli, Castelli *et al*. [35] demonstrated that these stimuli also engage brain regions associated with mental state reasoning and social cognition (see also [36]). Social context can also lead to mental state reasoning if stimuli are arranged in a manner that makes a moving object look like a social agent (such as an ice skater) rather than an inanimate object (like a spinning top [37]). Finally, the same movie footage of social interactions engages person knowledge networks more if real video footage is viewed rather than modified versions that have been made to appear ‘cartoonish’ [38]. Together, this work suggests that stimulus cues alone can provide an input to human-like mental state and animacy judgements.

### (d) Knowledge cues to human animacy

Knowledge cues to animacy are based on beliefs about an agent's animate origins and can be task instructed or task independent [39]. These can be considered ‘top-down’ cues that are driven by prior information about the stimulus, rather than by the visible form and motion cues. The impact of knowledge cues can be seen most clearly when visually identical stimuli are encountered across different conditions, which vary knowledge about the agent's humanness. Thus, any differences in cognitive or brain function are cued by information that is independent to the stimulus.

A growing body of behavioural evidence supports the notion that beliefs about humanness influence social perception and interaction [39–44]. For example, Liepelt & Brass [45] used an automatic imitation task and found that participants showed stronger evidence of motor priming when movements were thought to be made by a human rather than a wooden hand. Using simplified moving dot stimuli, Stanley *et al*. [41,42] showed increased behavioural interference together with reports of stimuli appearing more human-like when participants believed the stimuli originated from real human movement compared to computer-generated movement. Finally, using a manipulation where participants were required to coordinate their actions with a physically present humanoid robot, Stenzel *et al*. [43] found that participants were more likely to represent the robot's action if they believed that the robot's behaviour was based on a biologically inspired neural network than when it was based on a computer program.

Neuroimaging research has also varied knowledge cues to human animacy. Seminal fMRI studies of theory of mind used the same stimuli for both ‘human’ and ‘computer’ conditions, and varied participant instructions. The instruction ‘you are playing with a human’ gave rise to robust activation in the person knowledge network [46,47]. That is, the identical stimulus increasingly activated social brain regions when participants believed it originated in another person, not a computer.

### (e) Combined stimulus and knowledge cues to human animacy

Few studies have directly compared stimulus and knowledge cues to human animacy. Press *et al*. [28] showed that stimulus cues to animacy override knowledge cues when imitating hand actions. By contrast, Stanley *et al*. [41] showed that knowledge of how a moving dot was made (human versus computer-generated) dominated perception of animacy compared to its motion properties. Klapper *et al*. [40] showed that both types of cue influence imitation of hand actions. Moreover, fMRI results from the study by Klapper and co-workers showed that right TPJ was engaged more during an automatic imitation task when both stimulus *and* knowledge cues to human animacy were present than when only one or neither cue to human animacy was present [40]. This result supports the view that right TPJ may be particularly sensitive to controlling interactions with human agents [48,49].

A neuroimaging study by Stanley *et al*. [42] manipulated both types of cue by investigating passive observation of point-light animations. Point-light stimuli typically consist of a sequence of moving dots, representing several joints on an actor's body, which give the appearance of human biological motion [50]. This study found that knowledge of human animacy engaged mPFC more than knowledge that the stimuli were computer-generated. By contrast, human-like movement did not engage social brain circuits more than less-human movement. While emerging evidence suggests instances when both stimulus and knowledge cues influence social perception and cognition, the conditions and parameters that lead to these biases remain largely unknown.

### (f) Summary and the current study

Evidence suggests widespread cortical engagement of distinct social brain circuits for detecting and recognizing aspects of human animacy during social interactions. Stimulus and knowledge cues to human animacy engage person perception, action observation and mental state reasoning networks. The picture to date remains far from clear, but there appears to be some kernel of truth to the suggestion that a mechanism of self-similarity or ‘like me’ may operate across these studies. Many questions remain unanswered, however. A growing number of studies show indifferent or opposite brain or behavioural responses to those consistent with a theory based on self-bias. Moreover, few neuroimaging studies have directly compared stimulus and knowledge cues to human animacy in the same experiment to tease apart their relative contributions to detection and recognition of other humans. Indeed, only one other study to date has investigated action perception in this manner and this study did not present visible human features, such as faces or body parts, but instead used point-light displays of simple actions [42]. Hence, it remains unclear how perception of action is influenced by cues to human animacy, particularly when physical form cues are visible.

The current study, therefore, directly compares stimulus and knowledge cues to human animacy during the observation of agents interacting with objects. Face and body cues are manipulated as well as beliefs about the origins of such actions. By doing so, we are able to investigate which cues to animacy dominate perception of action as well as how these cues engage social brain circuits. To support the ‘like-me’ hypothesis, we would expect greater engagement of brain regions implicated in action observation [25,29], mentalizing [42,46,47] and person perception [25,26] when stimulus or knowledge cues to human animacy (or both) are present. However, as a number of recent studies suggest [21,30–33], we might also find that parts of the social brain are not solely tuned to preferentially respond to cues that are ‘like me’. Thus, the current study will provide novel insights into aspects of the social brain that are more or less responsive to features of an agent that are ‘like me’ through careful manipulation of stimulus and knowledge cues to human animacy.

## 2. Material and methods

### (a) Participants

Twenty-nine physically and neurologically healthy young adults were recruited from the fMRI Database of the Max Planck Institute for Human Cognitive and Brain Sciences (Leipzig, Germany). All were monetarily compensated for their involvement and provided written informed consent in line with procedures set forth by the local ethics board. Six participants were excluded from the final analyses due to not believing the cover story (see *Behavioural Procedure and Task*). The final sample included 23 participants (14 women, nine men; *M*_age_ = 26.41 years, s.d. = 3.02 years) who believed the cover story. All participants were native German speakers and right handed as measured by the Edinburgh Handedness Inventory [51].

### (b) Stimuli

**Figure 2.**
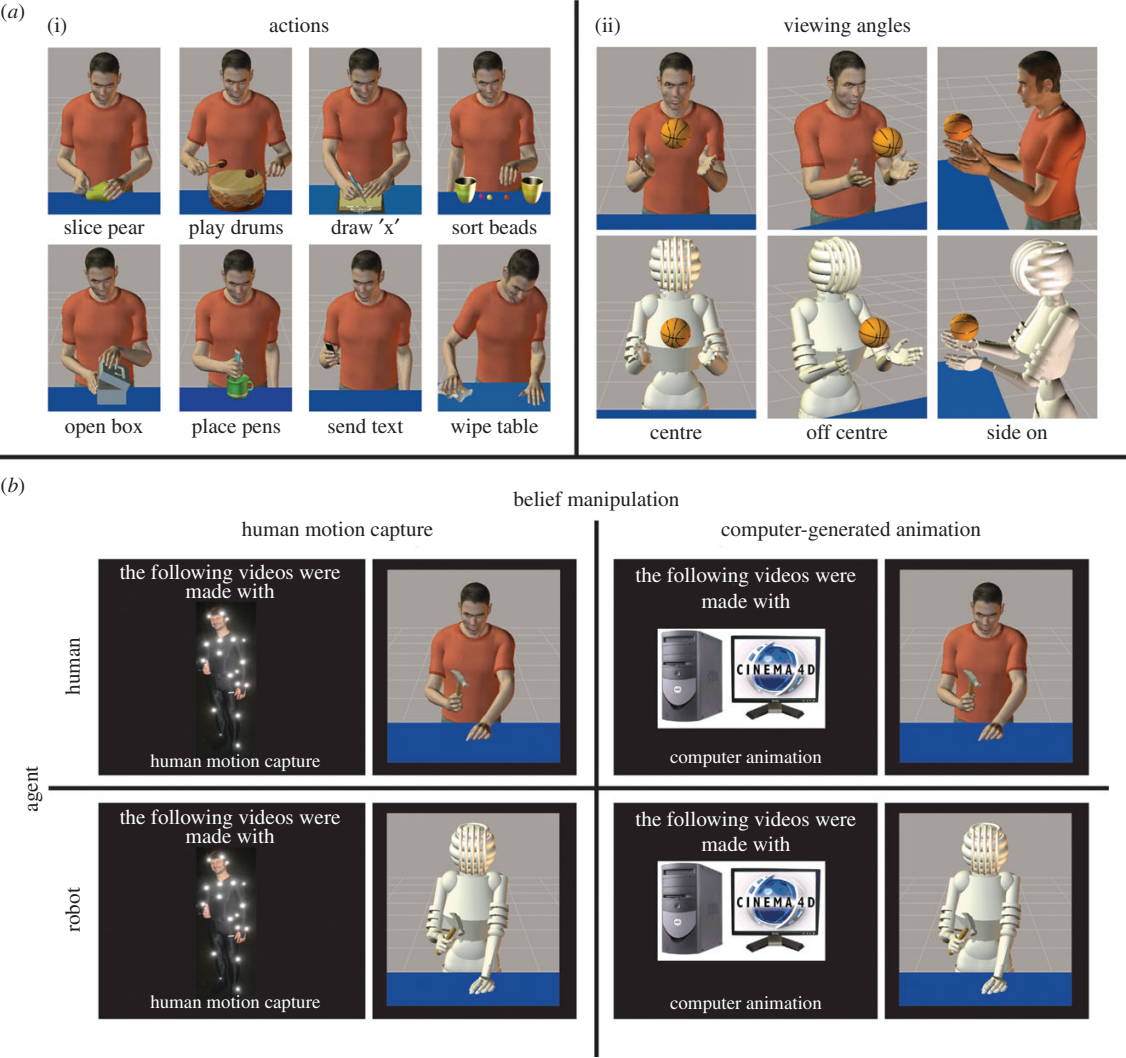
Details of experimental materials and design. (*a*)(i) Eight of the 10 actions featured in the stimuli set (the remaining two, ‘toss ball’ and ‘hammer nail’ are seen in (*a*)(ii) and in (*b*)). (*a*)(ii) The three viewing angles each action was ‘filmed’ from, to create a larger, richer stimulus set. (*b*) The 2 × 2 factorial design that enabled investigation of bottom-up features (whether the agent looked like a human or robot; rows of design) as well as top-down features (whether participants were told the videos were created using human motion capture or computer animation; columns of design).

Stimuli were created using Poser 7 three-dimensional animation software (SmithMicro Software Inc, Santa Cruz, CA, USA) and featured 10 object-directed actions (figure 2). Each video lasted 5 s. To create the stimuli, a human actor was first filmed performing each action, and these videos served as a model for creating the Poser videos. Each action was mapped onto two different avatars: a human male and a custom-designed robot (figure 2). Each action was ‘filmed’ from the waist upwards and from three different angles: centre, off centre and from the side (see right panel of figure 2*a*). These procedures yielded 60 videos in total (10 different actions × 2 different agents × 3 different viewing angles).

### (c) Belief manipulation

In order to manipulate knowledge cues to human animacy, participants were told the current study was commissioned by a major German film studio for the purpose of examining how the human brain processes two cutting-edge animation techniques: human motion capture and computer-generated keyframe animation. Before taking part in the experiment, participants watched a 10-min custom-made and professionally produced ‘documentary’ that explained human motion capture and computer keyframe animation techniques in detail (see also [40]). Specifically, participants learned that human motion capture involves recording real human movement via sensors that are attached to the body, whereas computer-generated keyframe animation involves a computer algorithm that fills in intermediate frames of a movement between predefined start and end positions. To further induce believability, the Poser stimuli used in the actual experiment were briefly seen in several parts of the cover story documentary to reinforce the idea that both kinds of animation could lead to the types of stimuli observed in the present study. In reality, however, all stimuli used in the real experiment were made with computer keyframe animation (the technique used by Poser software), which closely approximates real biological motion. After watching the documentary, participants were asked whether they had understood how both techniques were used to animate avatars, and whether they had any questions about the techniques before the experiment started.

### (d) Behavioural procedure and task

Participants' task in the scanner was to carefully observe 240 video stimuli during one functional run (each of 60 videos was repeated four times in total during the experiment). The videos were blocked into groups of five (with each group of five videos featuring either the human or the robot avatar), and participants observed a total of 48 blocks of five videos containing equal numbers of each agent form/belief pairing. Before each block of five videos was played, a cueing screen appeared for 2 s that specified that the following videos were made either with motion capture or computer keyframe animation (figure 1*b*). The order of instruction screens and the individual actions that made up each series of five videos was pseudo-randomly assigned.

After each video, one of two questions appeared which participants were required to answer: either (i) how much did you *like* the video you just saw? or (ii) how *smooth* did you find the movement in the previous video? These questions were chosen for several reasons. First, we wanted to determine how stimulus and knowledge cues to human animacy influence perception of the stimuli at a behavioural level. Second, two questions were chosen so that participants could not anticipate the exact question they would be asked, which required them to maintain attention to the stimuli. Participants made their ratings on a 1–8 scale via a fibre-optic scanner compatible button box. Following scanning, participants completed a debriefing survey where they were explicitly asked whether they noticed anything of note about the stimuli, as well as what they believed the true goal of the study was. The six participants (of the original sample of 29 participants) who raised suspicions the stimuli seemed to be the same and only the instructions changed were excluded from the final sample. Upon completing this survey, all participants were told the true nature of the study and compensated for their time.

### (e) MRI acquisition

Functional neuroimaging was acquired using a Bruker 3 Tesla Medspec 20/100 whole-body MR scanning system, equipped with a standard birdcage head coil. Functional images were acquired continuously with a single-shot gradient echo-planar imaging sequence with the following parameters: echo time (TE) = 30 ms, flip angle = 90°, repetition time (TR) = 2000 ms, acquisition bandwidth 100 kHz. Twenty-four axial slices allowing for full-brain coverage were acquired in ascending order (pixel matrix = 64 × 64; FOV = 24 cm, resulting in an in-plane resolution of 3.75 × 3.75 mm^2^, slice thickness = 4 mm, interslice gap = 1 mm). Slices were oriented parallel to the bicommissural plane (AC-PC line). The first two volumes of each functional run were discarded to allow for longitudinal magnetization to approach equilibrium. An additional 813–830 volumes of axial images were collected. Geometric distortions were characterized by a B0 field map scan (consisting of a gradient echo readout (32 echoes, inter-echo time 0.64 ms) with a standard two-dimensional phase encoding). The B0 field was obtained by a linear fit to the unwarped phases of all odd echoes. Following the functional run and field map scan, 24 two-dimensional anatomical images (256 × 256 pixel matrix, T1-weighted MDEFT sequence) were obtained for normalization purposes. In addition, for each participant, a sagittal T1-weighted high-resolution anatomical scan was recorded in a separate session. The anatomical images were used to align the functional data slices with a three-dimensional stereotaxic coordinate reference system.

### (f) Behavioural data analysis

Behavioural responses to the smoothness and liking questions asked during the imaging task were combined to form a single dependent variable and were analysed with a 2 (Agent Form: human, robot) × 2 (Belief Manipulation: human motion capture, computer-generated animation) repeated measures ANOVA.

### (g) Imaging data analysis

Data were realigned and unwarped in SPM8 (Wellcome Department of Imaging Neuroscience, London, UK) and normalized to the Montreal Neurological Institute (MNI) template with a resolution of 3 × 3 × 3 mm. Slice timing correction was performed after realignment. Functional data were normalized to individual participants' T1 anatomical scans with a resolution of 3 mm^3^. All images were then spatially smoothed (8 mm). A design matrix was fitted for each participant, with each type of video (Human with Motion Capture instruction, Human with Computer Animation instruction, Robot with Motion Capture instruction and Robot with Computer Animation instruction), the belief manipulation instruction screen and the question/response period modelled as a boxcar function convolved with the standard haemodynamic response function. The imaging analyses were designed to achieve the following three primary objectives:

#### (i) Main effect of stimulus cues

First, we evaluated the main effect of visual cues to the socialness of an observed agent. To achieve this, we compared observation of actions performed by the human avatar to the robot avatar (human > robot), as well as the inverse (robot > human).

#### (ii) Main effect of knowledge cues

We next assessed the main effect of our belief manipulation. We evaluated brain regions more engaged when videos were believed to have a human origin (motion capture > computer animation), or when videos were believed to be computer-generated (computer animation > motion capture).

#### (iii) Interaction between stimulus and knowledge cues

The third set of contrasts examined the interactions between agent form and belief cues. The aim of these interaction analyses was to determine the extent to which brain regions associated with the action observation, mentalizing or person perception networks are sensitive to specific pairings of stimulus-driven and knowledge-based cues to human animacy. The first interaction contrast interrogated brain regions more engaged when viewing congruent agent/belief pairings more than incongruent pairings. An example of a congruent pairing would be a human agent paired with motion capture belief or a robotic agent paired with computer-generated belief, whereas incongruent pairings would feature a human agent paired with computer animation belief or the robotic agent paired with motion capture belief. The inverse interaction examined brain regions more engaged when viewing the incongruent agent/belief pairings compared to the congruent pairings.

All neuroimaging analyses were evaluated at the whole-brain level with a voxel-wise threshold of *p* < 0.005 uncorrected and *k* = 10 voxels [52]. Table 1 lists all regions that meet this threshold. To most clearly illustrate all fMRI findings, *t*-images are visualized on a participant-averaged high-resolution anatomical scan. Parameter estimates (beta values) were extracted and plotted for visualization purposes only for the two interaction analyses. Anatomical localization of all activations was assigned based on consultation of the Anatomy Toolbox in SPM [53,54].

**Table 1.** Main effects and interaction from whole-brain analyses. MNI coordinates of peaks of relative activation within regions responding to the main effects of agent, collapsed across instruction (a: observing a human compared to a robot perform an action; and b: observing a robot compared to a human perform an action), the main effects of belief manipulation, collapsed across agent (c: observing actions said to be made by human motion capture compared to computer-generated animation; and d: observing actions said to be made by computer-generated animation compared to human motion capture) and the interactions between agent form and belief manipulation (e: observation of congruent agent/belief pairings; and f: observation of incongruent agent/belief pairings). Results were calculated at a voxel-level threshold of *p* < 0.005, *k* = 10 voxels. Up to three local maxima are listed when a cluster has multiple peaks more than 8 mm apart. Entries in bold denote activations significant at the false discovery rate cluster-corrected level of *p* < 0.05. HF, human form; RF, robot form; MCB, motion capture belief; CGB, computer-generated belief.

	MNI coordinates						
region	BA	*x*	*y*	*z*	*t*-value	cluster size	*p*-value
*main effect: stimulus-based cues to socialness*							
(a) human > robot agent							
no suprathreshold clusters emerged from this contrast							
(b) robot > human agent							
L fusiform gyrus	**37**	**−24**	**−76**	**−11**	**8.20**	**1422**	**<0.001**
R fusiform gyrus	**37**	**27**	**−76**	**−11**	**7.34**		**<0.001**
L cerebellum lobule VIIa		**−21**	**−82**	**−20**	**7.18**		**<0.001**
L superior temporal gyrus	22	−45	−1	−8	3.65	26	0.001
L hippocampus	28	−30	−31	−2	3.24	10	0.002
*main effect: knowledge-based cues to socialness*							
(c) human motion capture > computer-generated animation belief							
R inferior occipital gyrus	19	27	−79	−17	4.14	39	<0.001
R inferior occipital gyrus	19	36	−82	−14	3.75		0.001
R fusiform gyrus	37	39	−67	−20	3.33		0.002
L precuneus	5/31	−6	−37	43	3.77	48	0.001
L middle cingulate cortex	5	−6	−49	46	3.42		0.001
R fusiform gyrus	36	33	−43	−8	3.48	27	0.001
L superior parietal lobule	7/39	30	−70	46	3.24	11	0.002
(d) computer-generated animation > human motion capture belief							
no suprathreshold clusters emerged from this contrast							
*interactions between stimulus- and knowledge-based cues to socialness*							
(e) congruent > incong pairings: (HF w/ MCB) + (RF + CGB) > (HF w/ CGB) + (RF w/ MCB)							
L posterior cingulate cortex	23	−3	−43	10	4.00	24	<0.001
midline mid cingulate cortex	24	0	−13	31	3.96	32	<0.001
L middle cingulate cortex	24	−3	−22	25	2.89		0.004
L middle cingulate cortex	24	−3	−4	19	3.54	24	0.001
(f) incongruent > cong pairings: (HF w/ CGB) + (RF w/ MCB) > (HF w/ MCB) + (RF + CGB)							
R inferior frontal gyrus	45	33	23	19	4.15	17	<0.001
R cerebellum lobule VI		6	−79	−23	3.70	15	0.001

## 3. Results

### (a) Behavioural data

**Figure 3.**
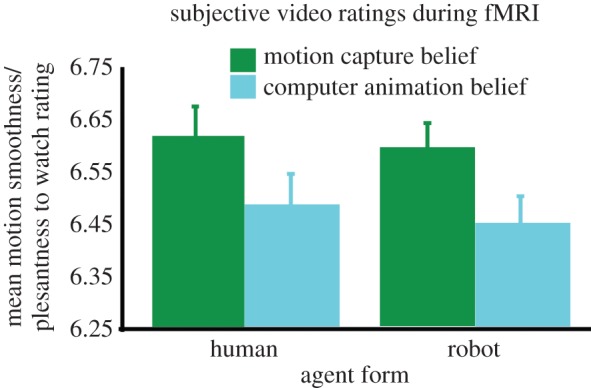
Behavioural data from fMRI task. Plots illustrate mean ratings reported by participants to questions interrogating how smooth participants found the movements or how much they enjoyed watching them. A main effect of belief was manifest, such that participants found those action videos they believed to originate from human motion capture techniques to be smoother and more enjoyable to watch than videos they believed to originate from computer-generated animation. No other main effects or interactions were observed.

During scanning, participants rated each video on how smooth they found the movement or how much they enjoyed watching it. Due to an error in the MATLAB code, it was not possible to separate ratings of liking and smoothness for the main experiment. However, a follow-up behavioural study was performed with 30 naive participants who performed the identical task with the same stimuli. These data showed that across all 120 stimuli/instruction pairings, ratings of liking and smoothness correlated at *r* = 0.53, *p* < 0.001. As prior work suggests that both questions tap into the same psychological construct (i.e. we tend to like movements more that are smooth [55], and participants' ratings of movement smoothness and liking strongly correlate in other experimental settings [56]), we considered it valuable to examine behavioural responses as a single combined variable. A repeated measures ANOVA revealed that participants rated movements they thought to be generated by human motion capture as significantly smoother and more pleasing to watch than videos they believed to be generated by computer animation, *F*_1,22_ = 21.28, *p* < 0.001 (figure 3). No main effect of agent (*p* = 0.39) emerged, nor was any interaction between belief and agent manifest in the data (*p* = 0.79). These data suggest that beliefs influence our dependent measure more than an agent's form.

### (b) Functional imaging data

#### (i) Main effects of stimulus cues

**Figure 4.**
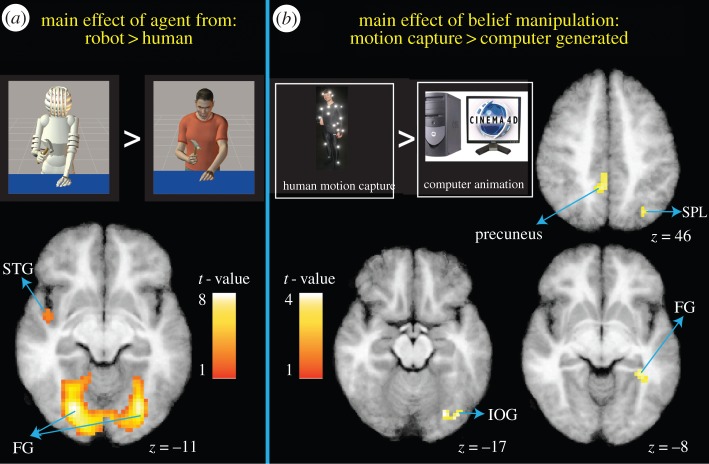
Main effects of agent form (stimulus) and belief manipulation (knowledge). Panel (*a*) illustrates brain regions more engaged when participants watched actions performed by a robotic avatar compared to a human avatar. Panel (*b*) shows brain regions more engaged when participants watched videos they believed to originate from human motion capture compared to computer animation. Full details of these findings are presented in table 1. STG, superior temporal gyrus; FG, fusiform gyrus; IOG, inferior occipital gyrus; SPL, superior parietal lobule.

The first imaging analyses investigated the extent to which visual cues to human animacy influence action perception. No suprathreshold clusters emerged from the human > robot form contrast. The inverse contrast (robot > human form) revealed engagement of bilateral ventral temporal and occipital cortices, which survived correction for multiple comparisons (*p* < 0.005, *FWE*-corrected), as well as *engagement of portions* of the left superior temporal gyrus and hippocampus (table 1*b* and figure 4*a*). Similar to findings reported by Cross *et al*. [32], this result suggests greater high- and low-level visual engagement when observing a robotic agent execute actions.

#### (ii) Main effect of knowledge cues

The next set of contrasts evaluated the impact of belief or knowledge cues to human animacy on action perception. The first contrast (human motion capture > computer keyframe animation belief) revealed activity within the right inferior occipital and fusiform gyri. While these brain regions did not survive correction for multiple comparisons, it is nonetheless of interest to note that the cluster located within the right inferior occipital gyrus closely corresponds to functional localizations of the OFA (less than 6 mm away [57]). Moreover, the peak of the cluster in fusiform gyrus is 14 mm away from an average peak location of this region when functionally localized, as reported by Spiridon *et al*. [58]. It should be noted, however, that the fusiform cluster identified in the present study is more anterior to most reports of the FFA. Clusters also emerged in the left precuneus, as well as the left superior parietal lobule also emerged from this contrast (table 1*c* and figure 4*b*). The response in the precuneus corresponds closely to responses typically found with a theory of mind localizer task based on comparing beliefs to physical stories [59]. The inverse contrast (computer keyframe animation > human motion capture) did not reveal any suprathreshold activations.

#### (iii) Interaction between stimulus and knowledge cues

The next set of analyses investigated the extent to which brain regions associated with social perception are influenced by the interaction of stimulus and knowledge cues to human animacy. The first interaction examined congruent pairings of agent and belief compared to incongruent pairings ((human form + motion capture belief) and (robot form + computer animation belief) > (human form + computer animation belief) and (robot form + motion capture belief)). Three uncorrected clusters emerged along the midline cingulate cortex, including middle and posterior cingulate cortices (table 1*e* and electronic supplementary material, figure A). The parameter estimate plots reveal evidence for crossover interactions for the two middle cingulate activations, while the interaction within posterior cingulate cortex appears to be driven most by a stronger response to the human agent being paired with motion capture instructions compared to computer animation instructions.

The inverse interaction evaluated brain regions more engaged when observing incongruent compared to congruent form and belief pairings ((human form + computer animation belief) and (robot form + motion capture belief) > (human form + motion capture belief) and (robot form + computer animation belief)). This contrast revealed two uncorrected clusters: one in the right inferior frontal gyrus, and a second in the cerebellum (table 1*f* and electronic supplementary material, figure B). For this interaction, it is of note that the interaction present within these two brain regions is driven by different stimuli (see parameter estimates in electronic supplementary material, figure B). Specifically, robotic agents paired with motion capture instructions seem to drive the cerebellar region most strongly, while the human agents paired with computer animation instructions drive the inferior frontal gyrus region most.

## 4. Discussion

Prior research has revealed that many different cues to human animacy engage brain networks associated with social cognition, while less is known about the relationship between these cues. In the present study, we used video stimuli featuring kinematically identical actions performed by a human or robotic agent and an elaborate belief manipulation to test the extent to which stimulus and knowledge cues to human animacy influence perception. Behaviourally, participants reported actions believed to originate from human motion capture to be smoother and more enjoyable to watch than those believed to have computer animation origins, while differences in agent form did not affect ratings. The neuroimaging findings echoed this pattern, with knowledge cues to human animacy showing subtle influence (at a liberal threshold) on brain circuits implicated in social cognition.

We failed to find evidence that visual cues to human animacy more strongly engage the action observation, person perception or theory of mind networks than visual cues to a robotic agent, as might have been predicted. In contrast, we found a robust, cluster-corrected area of activation spanning ventral temporal and occipital cortices when participants observed actions performed by a robotic compared to human-like agent. These findings raise questions about the role played by stimulus cues to human animacy, while also highlighting the influence of knowledge cues on social perception when perceiving identical agents and actions. Together, they provide new insights into the supporting neural architecture and behavioural consequences of social perception.

### (a) Belief about humanness influences perception, as shown by brain and behavioural responses

While some prior studies have failed to find evidence that belief about the human origins of a stimulus can impact perception [28], a growing body of evidence supports the notion that beliefs about humanness influence the way we perceive and imitate other agents [40–43,45]. Our results are consistent with these findings as participants were more likely to report actions supposedly originating from real human movements as smoother and more pleasing to watch (questions that tap into how natural or human-like an agent or action appears [56]). Our findings also fail to demonstrate that differences in agent form influence these ratings, which further suggests that knowledge cues can dominate stimulus cues in explicit evaluation of social features of an observed action [42]. A challenge for future behavioural research will be to systematically investigate how knowledge cues to animacy impact different facets of social cognition. To date, for example, perceptual and imitative processes have been studied separately, and the relationship between these key aspects of social cognition and knowledge cues to human animacy remains unexplored.

At the neural level, our findings provide some evidence that actions paired with a human- compared to computer-generated belief lead to greater engagement of brain regions associated with person perception and theory of mind. Specifically, portions of the right inferior occipital gyrus and fusiform gyrus responded more to the same stimuli when they were paired with human motion capture instructions. Both regions are located in close proximity to patches of cortex that are face selective including the OFA [57] and fusiform face [58] and body areas [60]. It is of note that these two brain regions associated with processing the human face were modulated in this instance by social knowledge, and not differences in stimulus-driven features.

Also important is the emergence of a cluster within the right precuneus from this same contrast. The precuneus is consistently implicated in theory-of-mind tasks and is believed to play a role in explicit belief processing [61,62]. If these results were to be replicated by future studies, they would suggest that parts of the social brain network involved in perceiving others' physical features and reasoning about others' minds are engaged when viewing agents whose actions are believed to have human origins. Revisiting the study by Stanley *et al*. [42], these researchers varied the motion parameters of point-light actions (ranging from veridical displays of the original action to completely scrambled versions of each action), and, as in the current study, they also varied instructions (human- or computer-generated). For the main effect of instructions (human > computer), and similar to the present study, Stanley and co-workers reported greater engagement of brain regions associated with mentalizing. Consistent with Stanley and co-workers' interpretation of this finding [42], we propose that based on believing that an agent is more human in nature, greater demands are placed on extracting relevant cues to support and evaluate this belief, changing the observer's perception of the social scene. In other words, it seems plausible that visual inputs are matched against a human template more in the human- than computer-belief condition. This process engages theory of mind and person perception in combination. This interpretation, however, remains speculative at this stage and will require further research to test it thoroughly.

### (b) Revisiting stimulus cues to human animacy and the action observation network's role in social perception

In contrast to a number of previous studies [27,39,63,64], we failed to find behavioural or brain-based evidence that stimulus cues to human animacy enhance action perception relative to non-human stimulus cues. Instead, we contribute further support, which survives correction for multiple comparisons, to a growing body of evidence that suggests that non-human stimulus cues can lead to the same or even an enhanced engagement of high- and low-level visual areas and the AON [21,30–33]. Specifically, we add to the evidence that social brain circuits including the AON are frequently indifferent to stimulus cues to human animacy.

Although visually salient differences between the human and robot avatar are apparent, the AON did not respond to this difference in the present study. An exploratory analysis of each stimulus form compared independently to an implicit baseline revealed that observing the human or robot agent in isolation resulted in widespread, robust engagement of bilateral AON, fusiform and occipitotemporal brain regions. The results of these simple contrasts help to rule out the possibility that the lack of findings in the human > robot contrast are due to a peculiarity of the human stimuli not engaging such brain networks on their own. The present findings could possibly be due to the fact that both agents executed the identical goal-directed actions (cf. [30,31]) or because the robot and human forms shared some features (i.e. a head atop a torso with two arms). Even though the human and robot forms were generated with the same CGI package, one potential reason the AON might have failed to discriminate between the agents might be because the human form was slightly less human than a video of a real person would be. Another possibility for why we found greater engagement of brain regions associated with person perception when observing a robot compared to a human could be that these brain regions are engaged to assimilate the robotic agent with a more familiar and predictable human template. A similar idea was discussed by Cross *et al*. [32] in light of finding more robust AON engagement when observing robotic compared to human-like actions. Recent work [65] lends tentative support to the idea that greater engagement of occipitotemporal brain regions when observing unfamiliar visual stimuli (such as the robotic actions in [32] and the robotic agents in the current study) might indeed be due to differences in predictability, as outlined by a predictive coding model of action perception [66].

Regardless of the reason for the absence of a difference in AON engagement observed between human and non-human stimulus cues in our study, the current findings suggest that the importance of a human-like form to social perception may have been overstated. Other factors such as top-down beliefs [42,45] and bottom-up kinematic information [27] also shape social cognition when perceiving and interacting with others [67,68]. Our data help to redress the balance of how much weight the AON assigns to self–other similarities on a form-based, visual level. Future research investigating perception of human animacy may explore which social brain mechanisms are specifically tuned to respond to the extent to which a stimulus is perceived as being ‘like me’, and what other complementary mechanisms might be at play [69,70]. Returning to the ‘like-me’ account of social cognition, the current findings contribute to this view by demonstrating that social brain circuits may be tuned to detect human animacy based on knowledge cues that signal an agent to be ‘like me’.

While we fail to find behavioural or imaging evidence demonstrating that visual cues to humanness influence social perception, it should be noted that exploratory further analysis of the human > robot form contrast (evaluated at *p* < 0.01, *k* = 10 voxels) revealed activity within the right temporoparietal junction, centred on coordinates *x* = 51, *y* = −37, *z* = 27. While this finding provides weak evidence that brain structures implicated in social cognition [59] might indeed be more engaged when observing human compared to robotic agents, we are reluctant to interpret this finding further due to the lack of statistical strength. The clearer message to emerge from the main effects of the present study is that top-down belief cues to human animacy shape social perception to a stronger degree than bottom-up visual form cues to human animacy, with stimuli paired with human beliefs associated with engagement of brain regions implicated in person perception and theory of mind.

### (c) Interactions between stimulus and knowledge cues to human animacy

The design of the present study enabled us to address how stimulus and knowledge cues to human animacy interact during action perception. Findings from the contrast comparing congruent with incongruent pairings of stimulus and knowledge cues failed to show modulation of the action observation, person perception or mentalizing networks. Instead, we report engagement of three uncorrected clusters spanning the middle and posterior cingulate cortex. However, as this finding was not predicted, we are reluctant to interpret it further. The result from the incongruent pairings interaction revealed an uncorrected cluster within the right inferior frontal gyrus located in a similar coordinate space to recent meta-analyses of the AON [9,11]. One simple interpretation of this finding, consistent with a rich literature on executive control, is that viewing incongruent pairings of agent form and humanness belief requires greater attentional control than when pairings are congruent [71]. Alternatively, it is possible that increased engagement of this sensorimotor brain region when viewing incongruent stimulus and knowledge pairings relates to increased demands on motor simulation mechanisms to reconcile human and artificial features of an observed agent. In order to evaluate this necessarily speculative interpretation, further research is required to replicate and more fully delineate how stimulus and knowledge cues to human animacy interact. If we take a step back and attempt to construct a broader view of how the current study's findings fit in to the wider literature on the biological substrates of social perception and social cognition, given that some findings do support the ‘like-me’ hypothesis [25–29], while others do not [30–33], and the fact that not all reported results survive correction for multiple comparisons, replication of these findings will be important for future progress towards understanding how we perceive animacy in other agents.

### (d) Multiple routes to socialness and considerations for social artificial agent design

The theoretical implications of the current study and research reviewed in this paper extend beyond the laboratory and serve to inform disciplines in addition to social cognition and neuroscience, including robotics. Over the past decade, individuals working to develop socially interactive artificial agents, including robots and avatars, are taking an increased interest in social cognition and social neuroscience research that examines the impact of ‘like-me’-ness on how we perceive and interact with such agents [72–75]. An ongoing goal for robotics designers has been to maximize the similarity of artificial agents to humans, in terms of appearance and movement (while perhaps attempting to circumnavigate the uncanny valley), in an attempt to make particular artificial agents as ‘like me’ as possible [76]. However, findings from the current study and considerations raised by related work suggest that how an agent is perceived as being ‘like me’ can take many forms and is not only dictated by how convincingly a robot looks or moves like a human. Pre-conceived beliefs about robots will impact their reception in the workplace, schools, care homes and other social settings, and will undeniably shape how effective human–robot interactions will be. Thus, human knowledge about and attitudes towards robots will need to be optimized as much as a robot's physical form and motion parameters. As such, roboticists and computer animators stand to benefit from further dialogue and collaboration with researchers investigating mechanisms of social perception and their consequences for social interaction.

## Supplementary Material

Supplemental Figure
