# Supplementary material for: The shaping of social perception by stimulus and knowledge cues to human animacy
Source: Philos Trans R Soc Lond B Biol Sci. 2016 Jan 19;371(1686):20150075. doi: 10.1098/rstb.2015.0075 (PMC4685521; doi:10.1098/rstb.2015.0075)
Supplement: Supplemental Figure [file rstb20150075supp1.pdf]

Supplementary Figure.
Interactions between stimulus and knowledge cues to human animacy

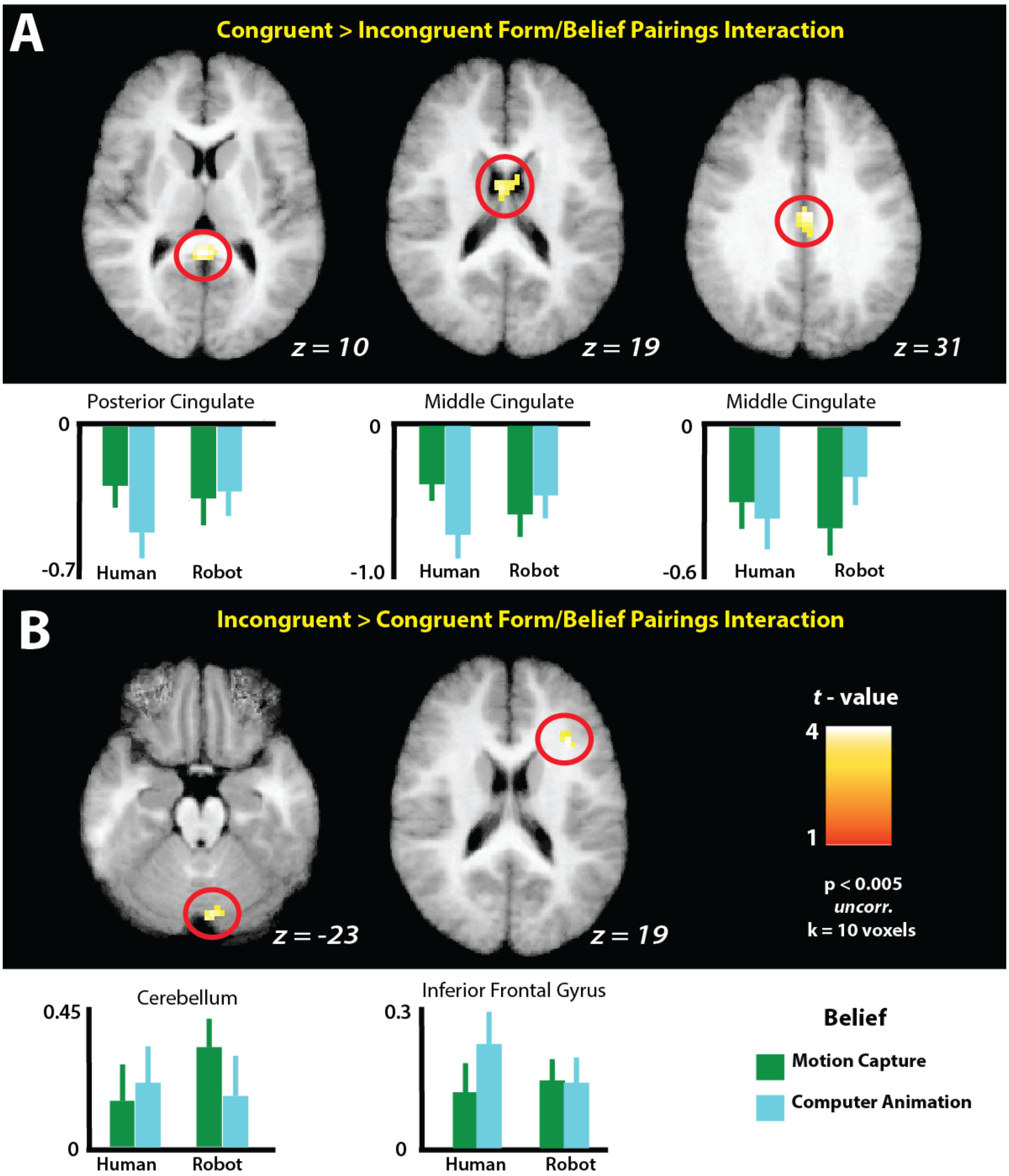

Panel A illustrates regions along midline cingulate cortex that emerged from the contrast comparing congruent form and belief pairings to incongruent pairings (see Material and methods in the main text for full explanation of pairings). Panel B illustrates activity within the cerebellum and inferior frontal gyrus that emerged from the interaction comparing incongruent to congruent pairings. Full details of these findings are presented in Table 1 of the main text.

Authors: Cross, Ramsey, Liepelt, Prinz and Hamilton
